# The rate and stability of mandibular block bone graft in recent 5 years

**DOI:** 10.1186/s40902-017-0118-0

**Published:** 2017-07-25

**Authors:** Jong-Cheol Park, Yun-Ho Kim, Hong-Seok Choi, Jong-Shik Oh, Sang-Hun Shin, Yong-Deok Kim

**Affiliations:** 1 Department of Oral and Maxillofacial Surgery, School of Dentistry, Pusan National University, Yangsan, Republic of Korea; 2 Dental Research Institute and Institute of Translational Dental Research, Pusan National University, Yangsan, Republic of Korea

**Keywords:** Implant stability, Mandibular block bone, Bone graft, ISQ, Impact factor

## Abstract

**Background:**

The purposes of the present study were to compare implant stabilities of mandibular block bone graft and bovine bone graft and to evaluate influencing factors for implant stability in mandibular block bone (MBB) graft.

**Methods:**

This retrospective study investigated 1224 cases and 389 patients treated by one surgeon in the Department of Oral and Maxillofacial Surgery of Pusan National University Dental Hospital (Yangsan, Korea) between January 2010 and December 2014. Proportions that MBB graft cases constitute in all implant restoration cases and in all bone graft cases were measured. Implant stability quotient (ISQ) values were achieved by the same surgeon before loading. The average ISQ values of the experimental groups were compared. In addition, ISQ values of influencing factors, such as age, sex, implant size, and implant placement site, were compared within the MBB group using Osstell^TM^ Mentor (Osstell®, Göteborg, Sweden). Paired *t* test and ANOVA were conducted for statistical analysis with a significance level of 0.05.

**Results:**

Fifty-five percent of all implant restoration cases performed bone graft while MBB cases constituted 34% of all implant restoration cases and 61% of all bone graft cases. Comparing ISQ values according to bone graft materials, the MBB group manifested sufficient implant stability by presenting comparable ISQ value to that of the experimental group without bone graft. Among the reviewed factors, females, mandibular molar regions, and implants in larger diameter displayed greater implant stabilities.

**Conclusions:**

Satisfactory implant stability was accomplished upon administration of MBB graft. Within the limitation of this study, gender, implant site, and implant diameter were speculated to influence on implant stability in MBB graft.

## Background

Tooth loss leads to change in bone resorption pattern and serious alveolar bone atrophy, ultimately risking successful implant restoration by reducing bone quantity and density. In order to resolve such problems, various surgical techniques have been suggested to reconstruct a severely atrophied jawbone. The traditional surgical methods with alveolar bone augmentation pose limitations on improving volumetric stability and implant stability. For patients with jawbone atrophy, an additional surgical procedure is inevitable to ensure sufficient amount of bone for implant installation. Herein, implant installation in autogenously grafted area has been suggested to reduce bone resorption and restore function.

Autogenous bone grafts can be commonly harvested from various donor sites including the calvarium bone and iliac bone [1–4]. Among the available sites, the mandibular bone has been widely adopted since it allows better accessibility, less scarring, and adequate harvest for implant installation [5]. Bone graft harvestings from the mandibular ramus and body bone have been more popular than symphysis bone harvesting which manifests higher morbidity including nerve injury and cosmetic concern [6, 7]. Nevertheless, few studies have studied the initial stability of implants installed after performing mandibular body bone graft, and a few studies have conducted statistical evaluation on a number of cases.

Therefore, the present study compared implant stability of mandibular block bone graft to that of bovine bone graft and investigated the influencing factors of implant stability in the patients who received mandibular block bone (MBB) graft.

## Methods

### Patients and materials

Of the 461 patients and 1639 cases of implant installation carried out by the same surgeon in the department of oral and maxillofacial surgery of Pusan National University Dental Hospital (Yangsan, Korea) between January 2010 and December 2014, 389 patients and 1224 cases were selected for this retrospective study.

The exclusion criteria were as follows:

1. Patients who did not measure ISQ
2. Patients whose follow-up was not possible for receiving prosthesis at different institutions
3. Graft materials that were neither MBB nor bovine bone (Bio-oss®, Geistlich AG, Wollhusen, Switzerland)

The fixtures used in this study were US II® (Osstem implant, Seoul, Korea), Solar® (Shinhung, Seoul, Korea), and SLActive® (Straumann, Basel, Swiss) whose surfaces were treated with sandblasting and acid etching.

This study was approved by the IRB review in Pusan National University Dental Hospital (IRB No. PNUDH-2016-036).

### Surgical procedures

**Fig. 1:**
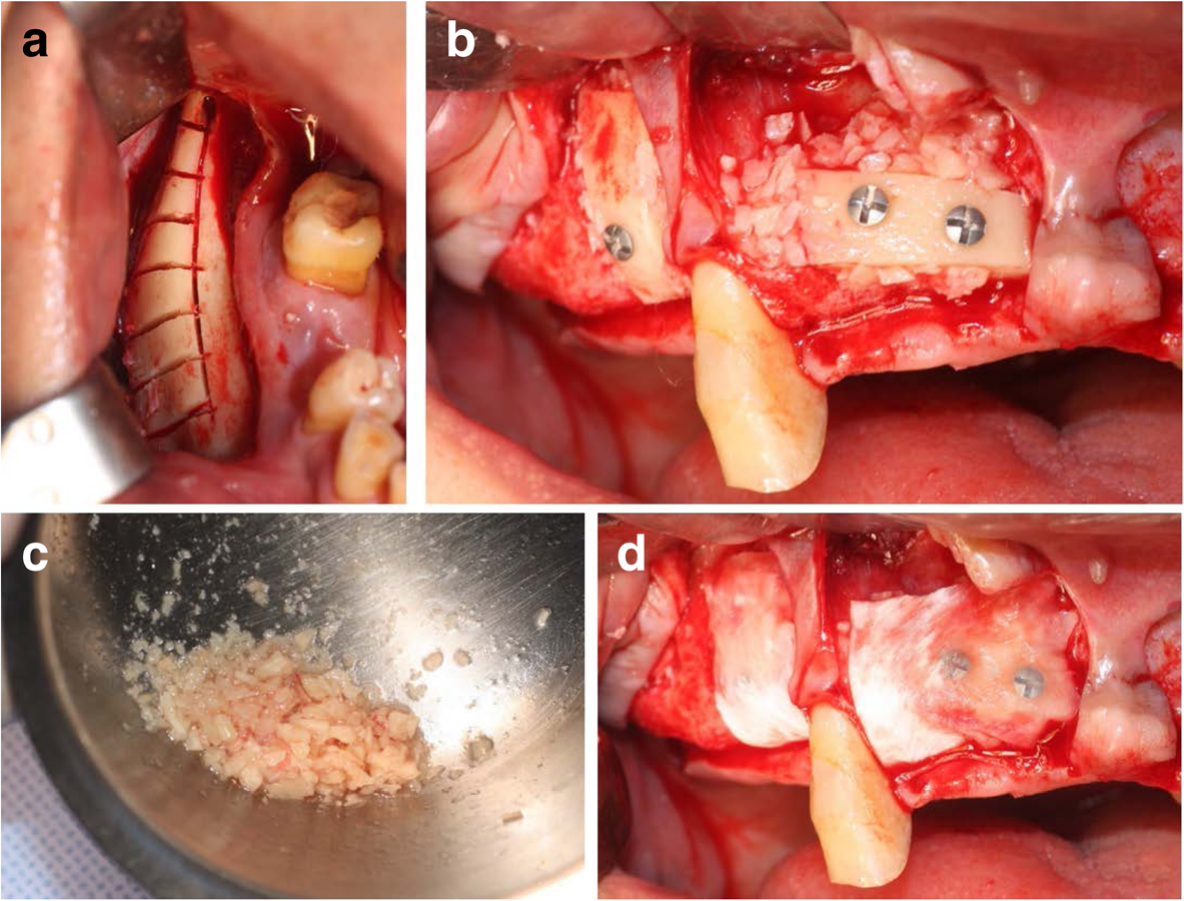
Procedure of MBB graft. **a** Donor site after sawing mandibular body bone. **b** Fixed block bone by lag screw technique. **c** Pariculated bone from harvested block bone. **d** Particulated bone positioned around fixed block bone with fibrin glue and covered with resorbable membrane

The surgical procedure of MBB graft included a harvest from cortical bone below the ramus bone which has been commonly utilized as harvesting site. After the harvested cortical block bone was fixed with a screw at expected implant placement site, cortical bone was partially particulated and mixed with fibrin glue around bone block and recipient site in order to reinforce the site. The grafted bone was completely covered with a resorbable membrane (OssGuide®, Bioland, Korea), and then, the site was sutured. Implant placement was generally performed after 4 to 6 months of healing period (Fig. 1) [8]. After the maxilla and mandible had 5–6 and 3–4 months of submerged period, respectively, ISQ values were examined prior to loading.

### Assessment of implant stability

The cases were categorized according to whether bone graft was performed or not. A total of three experimental groups including patients who did not receive bone graft, patients who received bone graft with bovine bone, and patients who received bone graft with MBB were investigated.

A ratio of MBB cases in all implant placement cases and that of MBB cases in all bone graft cases were assessed.

On purpose of evaluating implant stability, the average ISQ values were measured and compared among the experimental groups. In addition, ISQ values were analyzed within the MBB group according to sex, age, implantation site, and implant size, using Osstell^TM^ Mentor (Osstell®, Göteborg, Sweden).

### Statistical analysis

Paired *t* test and one-way ANOVA with the Bonferroni correction were used for statistical analysis with *p* value of <0.05 considered to be statistically significant. Analyses were conducted, using Microsoft Excel 2010® (Microsoft, Redmond, USA).

## Results

Six hundred seventy-four cases executed bone graft, taking up 55% of all 1224 implant restoration cases. Among bone graft cases, 416 cases were MBB graft, constituting 34% of all implant restorations and 61% of all bone graft cases. Two hundred thirty cases were bovine bone graft cases, and 28 cases included other bone grafts including iliac bone graft.

In the comparison of ISQ values according to bone graft materials (Table 1), the group without bone grafting presented the highest ISQ while the bovine bone group showed the lowest ISQ among the three experimental groups. In post hoc test, no significant difference was demonstrated between the group without bone graft and the MBB group. On the other hand, between the group without bone graft and the bovine bone group, the bovine bone group and MBB group showed apparent significance in ISQ values. Therefore, the MBB group demonstrated sufficient implant stability as its ISQ value was comparable to that of the group without bone graft.

**Table 1:** Comparison of ISQ value according to bone graft materials

	Without bone graft	MBB	Bovine bone
Case	550	416	230
ISQ	81.4 ± 6.55	80.87 ± 6.65	78.92 ± 6.96
		(*p* = 1.37 × 10^−5^)	

ISQ values were compared according to gender, age, implantation site, and implant size within the MBB group.

1. Gender
  Four hundred sixteen cases of mandibular block bone graft included 212 male patients and 203 female patients. The mean ISQ values of male patients and female patients were 80.17 and 81.71, respectively, demonstrating a significantly higher ISQ value in females (Table 2).

**Table 2:** Comparison of ISQ value according to gender and age

Age	<29	30~39	40~49	50~59	60~69	70~79	Total	ISQ by sex
								(*p* = 8.96 × 10^−3^)
Gender								
Male	15	12	74	57	40	14	208	80.17 ± 66.43
Female	8	36	35	68	53	6	198	81.71 ± 5.34
Total	23	46	109	125	93	20	416	
ISQ by age	80.13 ± 5.45	79.13 ± 8.89	81.35 ± 7.37	81.48 ± 6.06	80.97 ± 5.44	78.75 ± 5.9		Mean ISQ
(*p* = 0.21)								80.86 ± 6.65
2. Age
  The mean ISQ values for each age group were measured. However, no significant correlation between ISQ value and age group was observed (Table 2).
3. Implantation site
  As ISQ values were compared according to implant site, the ISQ values in mandibular region were significantly higher than those in maxillary region. In addition, mandibular molars displayed the highest ISQ values while maxillary molars manifested the lowest (Table 3).

**Table 3:** Comparison of ISQ value according to implantation site

Implantation site		Case	ISQ	
Maxilla	Anterior site	74	79.31 ± 6.28	(*p* = 3.92 × 10^−7^)
	Premolar site	85	80.92 ± 5.39	
	Molar site	113	78.88 ± 8.05	
Mandible	Anterior site	12	81.25 ± 4.16	
	Premolar site	51	81.98 ± 4.99	
	Molar site	81	84.25 ± 5.81
4. Implant diameter
  Comparing the mean ISQ values of each implant diameter, the mean ISQ values were increased with larger implant diameters (Table 4).

**Table 4:** Comparison of ISQ value according to implant size

Size of implants	Fixture diameter			Fixture height	
	<4mm	4 ~ 4.5mm	4.5mm<	<10mm	10mm≤
Case	26	292	98	139	277
ISQ	76.62 ± 4.86	80.1 ± 6.61	84.29 ± 5.78	80.5 ± 8.01	81.05 ± 5.85
	(*p* = 6.30 × 10^−10^)			(*p* = 0.4291)
5. Implant height
  There was no significant relationship between implant height and mean ISQ value (Table 4).

## Discussion

In the present study, MBB cases constituted 34% of 389 patients and 1224 cases in total and 61% of all bone graft cases performed by the same surgeon in the past 5 years from January 2010 to December 2014. More complicated implant restoration cases have been executed along with bone graft at Pusan National University Dental Hospital. This may be due to the limitations of local clinics, such as a deficient amount of bone graft materials.

Various methods have been introduced in order to evaluate initial implant stability. Recently, resonance frequency analysis (RFA), non-invasive method developed by the study of Meredith, has been the most accepted technique. In his in vivo study, Meredith connected a convertor onto implant to measure resonance frequency (RF) and ultimately to examine peri-implant bone [9, 10]. A number of studies employed RFA to investigate initial stability of implants, verifying the efficacy of the method [11–16]. Therefore, such non-invasive method of RFA can be qualified as an objective index to assess initial implant stability in the present study.

Mandibular body bone graft, which was used for MBB grafting in this study, and ramal bone graft share comparable properties except their harvest sites. In general, due to excellent accessibility and remote distance from inferior alveolar nerve, ramal bone graft has been chosen widely. However, body bone graft was adopted to contour more fittingly in the present study. Despite of numerous advantages of MBB graft as mentioned earlier, MBB graft faces disadvantages including potential damage on inferior alveolar nerve, insufficient available amount of harvest, and poor revascularization since it is mostly occupied with cortical bone [5, 17, 18]. On purpose of overcoming such weaknesses, CT analysis was performed to prevent nerve injury, and some cases with severe bone defect were excluded through judicious case selection [17]. Moreover, decortication was carried out in order to promote revascularization at recipient site [5].

In this study, implant stability of the MBB group was not significantly different from that of the group which did not receive bone graft. Furthermore, as comparing to the bovine bone group, the MBB group demonstrated greater implant stability. In general, implant placement was not challenging when performing GBR with bovine bone, and autogenous bone graft cases manifested less favorable environment for installing implants. The present study showed that satisfactory implant stability was achieved through autogenous bone graft even under unfavorable condition for implantation due to its outstanding bone formation and retention abilities.

Based on assessment of the MBB cases, the factors affecting implant stability included gender, implant placement site, and implant diameter. On the other hand, age and implant height demonstrated no significant influence on implant stability.

The influence of gender on implant stability has been controversial, but the present study showed better implant stability in females than in males. This may due to different habits and tendencies of each sex. In particular, smoking tendency, which is one of the major contributors of implant failure, is generally higher in males, and Sverzut et al. reported that male smokers tend to display higher implant failure [19].

Many studies have verified the effect of age on implant stability [20, 21], but a few studies have assessed a relationship between age and implant stability in autogenously grafted area. In the present study, there was no significant association between age and implant study in the MBB cases. Therefore, further studies are necessary to investigate with more specimens and cases.

Among the various implant placement sites adopted in this study, mandibular posterior regions showed the highest implant stability and maxillary posterior regions displayed the lowest. Esposito et al. reported a higher implant failure in maxilla than in mandible [22]. Furthermore, Steenberghe et al. revealed that the posterior region in maxilla particularly manifested more failures [23]. The present study also observed similar results that may be due to differences in bone quality and quantity between maxilla and mandible. However, further studies are required to study a relationship between implant site and implant stability after grafting autogenously, MBB graft in particular.

In many cases, larger implant diameter achieves greater implant stability [24]. Smaller implant diameter tends to risk successful implant restoration for retaining less support against occlusal load. However, when implant diameter exceeds the standard size, implant stability declines [25]. Implants of more than 5 mm in diameter are commonly chosen on purpose of rescuing implants of 4 mm when poor bone quality seems to be responsible for implant failure. Nevertheless, smaller implant diameters exhibited lower ISQ values in this study. Although a research has shown that narrow implants can achieve acceptable stability in edentulous posterior area [26], the present study demonstrated lower ISQ values for narrower implants. This may be since MBB graft allowed sufficient bone quality and quantity in most of the cases.

In addition, longer implant height provides satisfactory implant stability while shorter implant height generally increases implant failure rate [24]. Some studies have reported that sufficient stability can be achieved with a length of 8 mm or more [27]. However, the present study showed no significant correlation between implant height and ISQ value. Furthermore, implants less than 8 mm in height were barely used in this study. Through MBB graft, adequate bone height was achieved successfully, requiring no excessively short implants.

## Conclusions

This study has demonstrated higher bone graft rates at the present hospital than local dental clinics, and MBB graft constituted majority of the bone grafts performed at the hospital. Furthermore, satisfactory alveolar ridge augmentation with excellent quantity and quality of alveolar bone was acquired successfully, and adequate implant stability was accomplished through MBB graft. Within the limitation of this study, it seemed crucial to cautiously consider gender, implant site, and implant diameter when deciding implant placement after MBB graft.
